# A Descriptive Analysis of the Scientific Literature on Meteorological and Air Quality Factors and COVID‐19

**DOI:** 10.1029/2020GH000367

**Published:** 2021-07-20

**Authors:** Amanda V. Quintana, Meredith Clemons, Krista Hoevemeyer, Ann Liu, John Balbus

**Affiliations:** ^1^ ICF International ‐ U.S. Global Change Research Program Washington DC USA; ^2^ George Washington University Washington DC USA; ^3^ Des Moines University ‐ U.S. Global Change Research Program Des Moines IA USA; ^4^ National Institute of Environmental Health Sciences Bethesda MD USA

**Keywords:** climate, weather, public health, COVID‐19

## Abstract

The role of meteorological and air quality factors in moderating the transmission of SARS‐CoV‐2 and severity of COVID‐19 is a critical topic as an opportunity for targeted intervention and relevant public health messaging. Studies conducted in early 2020 suggested that temperature, humidity, ultraviolet radiation, and other meteorological factors have an influence on the transmissibility and viral dynamics of COVID‐19. Previous reviews of the literature have found significant heterogeneity in associations but did not examine many factors relating to epidemiological quality of the analyses such as rigor of data collection and statistical analysis, or consideration of potential confounding factors. To provide greater insight into the current state of the literature from an epidemiological standpoint, the authors conducted a rapid descriptive analysis with a strong focus on the characterization of COVID‐19 health outcomes and use of controls for confounding social and demographic variables such as population movement and age. We have found that few studies adequately considered the challenges posed by the use of governmental reporting of laboratory testing as a proxy for disease transmission, including timeliness and consistency. In addition, very few studies attempted to control for confounding factors, including timing and implementation of public health interventions and metrics of population compliance with those interventions. Ongoing research should give greater consideration to the measures used to quantify COVID‐19 transmission and health outcomes as well as how to control for the confounding influences of public health measures and personal behaviors.

## Key Points

- The 61 peer‐reviewed epidemiological studies described in this analysis used a wide variety of methods to explore associations between meteorological and air quality factors and COVID‐19 health outcomes associated with the pandemic
- The majority of studies did not adequately account for temporal and geographic uncertainty when using proxy measures of SARS‐COV‐2 transmission, such as laboratory testing reports
- Future studies should appropriately account for assumptions about transmission timing and control for a baseline set of confounders

## 1 Introduction

On March 11th, 2020, the novel coronavirus disease, COVID‐19, was declared a global pandemic. COVID‐19 is transmitted from human to human, primarily through respiratory aerosols and droplets, and is a respiratory illness that may be influenced by climatic conditions similar to how other respiratory viruses exhibit seasonality (Dalziel et al., 2018; Huang, Wang et al., 2020). Early studies of the COVID‐19 pandemic have suggested that temperature, humidity, ultraviolet radiation (UV), and other meteorological factors have an influence on the transmission and viral dynamics of the disease possibly by inactivating the SARS‐CoV‐2 virus or affecting human defense against viral infection in the respiratory airways (Eccles, 2002; Soane et al., 2001). A systematic review by Mecenas et al. (2020) examined studies characterizing seasonal and meteorological factors during the course of the pandemic from late 2019 to March 24th, 2020. The authors concluded that warm and wet climates reduced the spread of COVID‐19 but noted the certainty of the evidence was low and variability in transmission was not well‐explained by meteorological factors. Shakil et al (2020) completed a critical analysis of studies that looked at the impacts of COVID‐19 by and on environmental factors. The authors noted that many studies examining the influence of meteorological factors and air quality on COVID‐19 used descriptive or correlative analytic methods, and few accounted for non‐linearity in the associations between meteorological factors and disease outcomes. The authors raised concerns about potential confounding factors such as population mobility or age in looking at associations with meteorological factors. This study aims to build upon these previous literature reviews by incorporating more recent, peer‐reviewed literature and by focusing in greater detail on strong epidemiological methods of COVID‐19 studies.

## 2 Materials and Methods

### 2.1 Literature Search

This review makes use of a search conducted by the National Oceanic and Atmospheric Administration (NOAA) Library on meteorological and air quality factors and COVID‐19 of the existing literature through July 3. The Library used the following search criteria: (“COVID 19” OR “SARS‐CoV‐2” OR “2019‐nCoV” OR “Wuhan coronavirus”) combined with various meteorological variables including (“Humidity” OR “wind speed” OR “air pressure” OR “meteor*” OR “dew point” OR “precipitation” OR “rainfall” OR “pollut*” OR “diurnal temperature” OR “weather” OR “season*” OR “air quality” OR “nitrogen dioxide” OR “latitude” OR “UV index” OR “cloud cover” OR “temperature region*” OR “elevation” OR “Nitrogen Dioxide”). The databases used were Web of Science, Science Direct, PubMed, Dimensions, Lens, arXiv, bioRxiv, medRxiv, SSRN, and Google Scholar.

### 2.2 Study Selection and Variable Extraction

**Figure 1:**
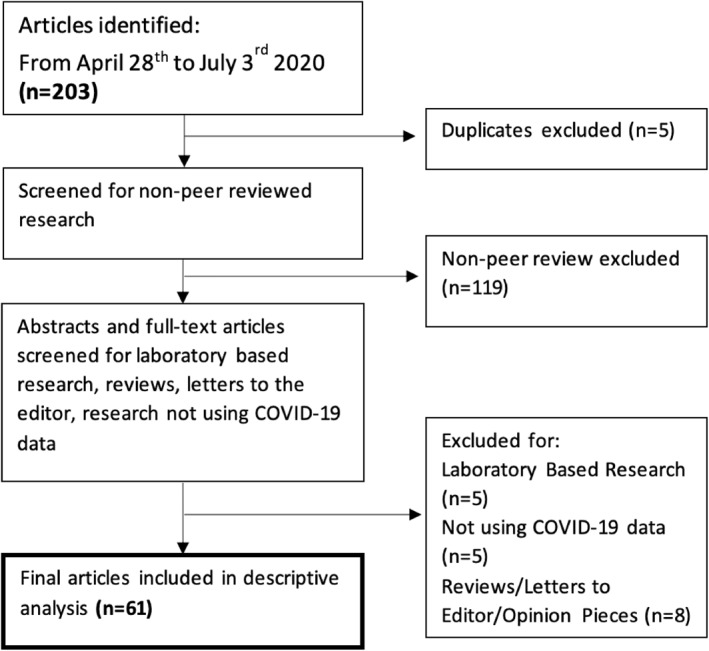
Eligibility criteria flow chart. Source: Authors.

Of the 203 English language studies obtained from the NOAA search, 61 observational, epidemiological, original, peer‐reviewed research articles were selected for analysis (Figure 1) **(**Adhikari & Yin, 2020; Ahmadi et al., 2020; Asyary & Veruswati, 2020; Auler et al., 2020; Bashir, Ma, Bilal, Komal, Bashir, Tan, & Bashir, 2020; Bashir, Ma, Bilal, Komal, Bashir, Farooq, et al., 2020; Benedetti et al., 2020; Biktasheva, 2020; Briz‐Redón & Serrano‐Aroca, 2020; Byass, 2020; Coccia, 2020; Del Rio & Camacho‐Ortiz, 2020; Demongeot et al., 2020; Fareed et al., 2020; Fattorini & Regoli, 2020; Goswami et al., 2020; Guasp et al., 2020; Gunthe et al., 2020; Gupta, Banerjee, & Das, 2020; Gupta, Raghuwanshi, & Chanda, 2020; Haghshenas et al., 2020; Huang, Huang, et al., 2020; Iqbal et al., 2020; Jahangiri et al., 2020; Jiang et al., 2020; Jüni et al., 2020; Li, 2020; Liu et al., 2020; Livadiotis, 2020; Ma et al., 2020; Méndez‐Arriaga, 2020; Menebo, 2020; Mollalo et al., 2020; Ogen, 2020; Paez et al., 2020; Pani et al., 2020; Passerini et al., 2020; Pequeno et al., 2020; Pirouz, Haghshenas, Haghshenas, & Piro, 2020; Pirouz, Haghshenas, Pirouz, et al., 2020; Prata et al., 2020; Qi et al., 2020; Rosario et al., 2020; Runkle et al., 2020; Şahin, 2020; Sajadi et al., 2020; Sarmadi et al., 2020; Scafetta, 2020; Shahzad et al., 2020; Shi et al., 2020; Sobral et al., 2020; Tobías & Molina, 2020; Tosepu et al., 2020; Ujiie et al., 2020; Ward et al., 2020; Wu et al., 2020; Xie & Zhu, 2020; Xu et al., 2020; Zhu et al., 2020; Zoran et al., 2020a; Zoran et al., 2020b). Three individuals divided the 61 studies for review, and from each selected study, extracted information on COVID‐19 health outcomes, meteorological and air quality variables analyzed, their data sources, confounding variables controlled for, and unmeasured confounders acknowledged. Where appropriate, variables were binned into higher‐order descriptors for clarity and simplicity. For example, different measures of temperature (maximum, minimum, etc.) were binned as “temperature”. Additionally, information about significant associations and statistical methods was extracted. Details on the individual terms within each bin are included in Supporting Information (Data Set S1).

### 2.3 Approaches Used for Data Extraction

#### 2.3.1 Health Variable

All health variables were binned into one of four categories for this analysis. Categories included daily new cases, cumulative cases, daily deaths, and cumulative deaths. Health variables that could not be binned into one of these categories were labeled “other”.

#### 2.3.2 Meteorological/Air Quality Factor

Variables were binned into one of six categories which included temperature, humidity, precipitation, wind speed, air quality, and UV radiation. Factors that could not be binned into one of these six categories were labeled “other”.

#### 2.3.3 Geographical Scale

Studies were categorized based on the type of the geographic region examined. Location categories included city, state/province, country, or global – which included 30 countries or more or two continents.

#### 2.3.4 Lagged Exposures

Studies that accounted for the incubation period by measuring exposure variables lagged by a specified time frame were identified.

#### 2.3.5 Statistical Approach

Analyses were binned in one of three categories: correlation or simple comparisons among groups; regression; and advanced regression. Analyses that did not fall into these categories were labeled as “other”.

#### 2.3.6 Statistical Significance

This study categorized statistically significant associations based on the main analysis conducted in a study. For those studies that provided a *p*‐value, the most adjusted results with *p*‐values <0.05 were considered significant. For studies that reported a ratio, a 95% confidence interval that excluded the null (1) was considered significant.

#### 2.3.7 Potential Confounding or Modifying Variables

Variables included as potential confounding or modifying variables in statistical analysis models were extracted in the following categories: age, gender, population density, pandemic phase (Coninx et al., 2009), comorbid health conditions, population movement, and socioeconomic status. Those studies that did not control for potential confounders or modifiers were included in the “none” category, while those studies that controlled for a variable that did not fall into the designated categories were included in the “other” category.

#### 2.3.8 Unmeasured Contextual Factors

We additionally noted factors that were identified in the study (often within the discussion section) as having a likely influence on COVID‐19 transmission but that were not able to be quantified or included in the statistical analysis.

## 3 Results

### 3.1 Description of Study Categorization (Tables 1–4)

#### 3.1.1 Number of Health Variables in Studies and COVID Data Sources Used in Studies

Most of the 61 studies **(**Table 1 **)** assessed associations with either new COVID‐19 cases (*n* = 34) or cumulative cases (27). Ten examined cumulative deaths, while six used daily death rates. Of the 61 studies, 20 studies looked at more than one health variable, two of which looked at three health variables. Most studies used COVID‐19 data that were collected by a local health authority. Thirteen studies used “other” data sources that included commercial and other private sector sources; few studies did not specify the source of their COVID‐19 data.

**Table 1:** Number of Health Variables in Studies and COVID Data Sources Used in Studies

COVID‐19 health outcome	Count	Data source	Count
Daily new cases	34	WHO	4
Daily deaths	6	Johns Hopkins	7
Cumulative cases	27	Local/National Health Authority	33
Cumulative deaths	10	Not specified	4
Other	6	Other	13
Total	83		61

#### 3.1.2 Meteorological/Air Quality Factors and Data Sources

Of the meteorological and air quality variables (Table 2), temperature was the most common factor observed in 52 of the 61 studies. Other common meteorological variables included humidity, precipitation, and wind speed. Air quality was observed in 15 of the studies. The majority of studies analyzed more than one environmental factor. The ‘other’ category included meteorological and air quality factors such as solar radiation, cloud cover, vertical airflow, sunlight exposure, wind direction, dew point, and atmospheric pressure field. Twenty studies used a national meteorological service source or a commercial/private data source. Two studies relied on data from two different sources.

**Table 2:** Meteorological/Air Quality Factors and Data Sources (*n* = 61 Studies)

Factor	Count	Data source	Count
Temperature	52	Global/Regional data set	11
Humidity	37	National Met Service	20
Precipitation	19	Local data source	6
Wind speed	18	Commercial/Private	20
Air quality	15	Not specified	6
UV	3	Total	63
Other	22		
Total	166		

#### 3.1.3 Type of Statistical Analysis and Use of Confounding Variables

The 61 studies used a range of statistical approaches (Table 3). The majority used regression methods, which included studies that used both linear and multivariate regressions. Eight used more advanced methods, which included multilevel, random effects or generalized linear mixed models, spatial analyses, seemingly unrelated regression, Poisson regression with distributed lag linear and nonlinear models, and artificial intelligence modeling. The ‘other’ category included distributions, receiver operating characteristic curves (ROCs), and simple data plots.

**Table 3:** Statistical Analysis

Statistical analysis	
Correlation	20
Regression	27
Advanced regression	8
Other	6
Total	61

The majority of studies failed to account for sources of bias in the study design, such as the presence of a third factor that potentially distorts or modifies the relationship between exposures and health outcomes. Of the 61 studies, 39 studies did not control for potentially confounding or modifying variables, leaving 22 studies that looked at one, two, or more potential factors (Table 4). The ‘other’ category included variables such as elderly income, number of companies in a province, health‐seeking behavior, and climatic factors. A few studies alluded to unmeasured confounders in their discussions of their analyses but did not collect information on them (data not shown).

**Table 4:** CVs Used and Counts of CVs Within Studies

Confounding variable	Count
Age	4
Gender	0
Population density	9
Pandemic phase	5
Health conditions	0
Migration	4
Socioeconomic status	1
None	39
Other	10
# Studies with 0 CVs	39
# Studies with 1 to 2 CVs	12
# Studies with >2 CVs	10
Total	61

Abbreviation: CVs, confounding variables.

### 3.2 Description of Study Results in Association With Specific Variables and Methodological Approaches (Tables 5–8)

#### 3.2.1 Statistical Associations by Meteorological and Air Quality Factors

Of the 61 studies reviewed, 43 examined multiple meteorological and air quality factors in their analyses (Table 5). As a result, a total of 166 associations were analyzed across the 61 studies. We report here the associations from those models within a study that used the greatest number of covariates. Temperature and humidity were most frequently analyzed, with 52 and 37 examinations of association, respectively. With the exception of temperature and air quality, most analyses did not find a statistically significant association between meteorological and air quality factors and health variables. “Other” factors examined included solar radiation, cloud cover, evaporation, altitude, latitude, dew point, and air pressure. “Other” factors with statistically significant associations included solar radiation and sunlight exposure, dew point, and latitude.

**Table 5:** Statistical Associations by Meteorological and Air Quality (Met/AQ) Factors

Met/AQ factor	Association	No association	Total
Temperature	30	22	52
Humidity	16	21	37
Precipitation	6	13	19
Wind speed	7	11	18
Air quality	9	6	15
UV	0	3	3
Other	12	10	22
Total	80	86	166

#### 3.2.2 Associations of Meteorological and Air Quality Factors by Type of Health Outcome Variable

A higher proportion of the 61 studies found significant associations between temperature and humidity and daily new cases and deaths, as opposed to cumulative health measures (Table 6). The majority of studies examining associations between air quality parameters and health outcomes also showed significant associations, but as seen in Table 6, most studies examined multiple air quality parameters. No single air quality measure showed a trend of more frequent associations. There were no associations found in the UV studies or for the “other” category of health variables, so they were excluded from the table.

**Table 6:** Statistical Associations by Health Variable & Met/Env Factor

	Association	No association	% Association
Daily new cases			
Temperature	18	13	58.1
Humidity	11	9	55.0
Precipitation	3	7	30.0
Wind speed	4	6	40.0
Air quality	6	2	75.0
Total	34	26	56.7
Cumulative cases			
Temperature	10	15	40.0
Humidity	6	13	31.6
Precipitation	2	9	18.2
Wind speed	4	9	30.8
Air quality	5	2	71.4
Total	27	48	36.0
Daily deaths			
Temperature	3	1	75.0
Humidity	3	1	75.0
Precipitation	1	0	100.0
Wind speed	0	1	0.0
Air quality	1	2	33.3
Total	8	5	61.5
Cumulative deaths			
Temperature	1	5	16.7
Humidity	1	5	16.7
Precipitation	0	2	0.0
Wind speed	0	3	0
Air quality	2	3	40
Total	4	18	18.2

Fifteen studies looked at the relationship between air quality and COVID‐19. Most studies looked at multiple air quality variables. The most common air quality measure was PM 2.5, followed by NO2, ozone, and PM10 (Table 7). The number of associations found (*n* = 28) was similar to those lacking an association (*n* = 25).

**Table 7:** Association With Specific Air Quality Variables (*n* = 15 Studies)

Air quality variable	No. of studies	Association	No association
PM 10	8	5	3
PM 2.5	10	4	6
Volatile organic compounds	1	0	1
SO_2_	5	3	2
CO	4	3	1
NO_2_	9	4	5
Ozone	9	6	3
Pb	1	1	0
Air quality index	5	1	4
Not specified	1	1	0
Total		28	25

#### 3.2.3 Statistical Associations by Meteorological and Air Quality Actor and Type of Analysis

The majority of the 61 studies used either “correlation or differences among groups'' analyses or “regression” analyses (Table 8). The use of “advanced analyses” for a given factor was limited, ranging from eight advanced analyses for temperature to one advanced analysis each for wind speed and air quality. Across the meteorological and air quality factors, analysis type “other”, which included distributions, plots, and ROC curves, was excluded from the table because statistical testing was not involved in the six studies. UV radiation was excluded from this table as only three analyses were reported, none of which found a statistically significant association with the health variable. “Other” meteorological factors were also excluded from the table. Twelve of 22 associations were statistically significant.

**Table 8:** Statistical Associations by Met/Env Factor and Type of Analysis

	Association	No association	Total
Temperature			
Correlation or difference between groups	11	5	16
Regression	16	8	24
Advanced analysis	3	5	8
Total	30	18	48
Humidity			
Correlation or difference between groups	2	10	12
Regression	13	4	17
Advanced analysis	1	4	5
Total	16	18	34
Precipitation			
Correlation or difference between groups	2	7	9
Regression	4	4	8
Advanced analysis	0	2	2
Total	6	13	19
Wind speed			
Correlation or difference between groups	3	6	9
Regression	4	4	8
Advanced analysis	0	1	1
Total	7	11	18
Air quality			
Correlation or difference between groups	4	2	6
Regression	5	1	6
Advanced analysis	0	1	1
Total	9	4	13

## 4 Discussion

The 61 peer‐reviewed epidemiological studies described in this analysis used a wide variety of data sources and methods to explore associations between air quality and meteorological factors and COVID‐19 outcomes. There were limitations regarding the COVID‐19 health data. Because the exact timing of person‐to‐person transmission of the SARS‐CoV‐2 virus is unavailable, a proxy measure of disease transmission must be used. In most studies, daily reporting of positive lab results (cases) was the proxy for the transmission event. However, this proxy measure is a problematic estimate of virus transmission due to uncertain and variable lag periods between initial infection and the reporting of lab results. In addition, the majority of studies used data from local or national health authorities, and different data sources have used varied data collection methods. Therefore, data collection can be inconsistent, making it difficult to compare studies. For example, WHO’s COVID‐19 database uses only “confirmed cases,” while the Johns Hopkins COVID‐19 dashboard includes “probable cases”. Additionally, other key factors, including the availability of tests and differential access to testing among populations, could exert strong confounding effects on the relationship between daily meteorological and air quality factors and disease transmission.

Studies differed in the type of COVID‐19 health outcomes examined. Most studies analyzed case data, either daily or cumulative, rather than death data. The selection of case versus death data often depended on the primary research question in the study. If the study was focused on disease transmission, case data would generally be used, while death data were more of a proxy for COVID‐19 severity. As noted in the results of this review, daily cases rather than cumulative cases were associated with more significant findings based on this relatively small sample of studies. While the reasons for this are unclear, studies using daily cases tending to examine the influence of daily fluctuations in meteorological factors in a given place, while studies using cumulative cases tending to make comparisons between places. This opens the possibility of unidentified potentially confounding factors associated with location, which could weaken the ability to find associations with meteorological factors. Should this distinction between studies using daily and cumulative cases remain, the reasons for differences should be studied more systematically.

Overall, the majority of studies failed to account for the timing between transmission of the SARS‐CoV‐2 virus and manifestation of COVID‐19 infection when analyzing meteorological and air quality data measurements and COVID‐19 health outcomes. Only 12 of the 61 studies examined lagged exposures to account for the incubation period. When studies assessed transmission, uncertainty and variability in the timing between the transmission event and the reporting of cases and deaths were not addressed in the analysis. It is important that future studies acknowledge the importance of lagged exposures and design data collection and statistical analyses accordingly.

In terms of evaluating the rigor of epidemiologic methods, this descriptive review of the literature reveals heterogeneity in the types of statistical methods used to analyze data and in the inclusion of appropriate potentially confounding variables. Twenty of the 61 studies relied only on simple correlations to conclude associations existed between air quality and meteorological factors and COVID‐19 health outcomes, yet correlations are not considered a rigorous statistical approach. More robust methods that account for multiple variables in an analysis, and even further, methods that address geographic or temporal dependence, are needed to improve the validity of results. Only around a third of the studies accounted for important potential confounders such as age, population density, and socioeconomic status. Factors such as these could be related to both exposure to meteorological and air quality factors as well to COVID‐19 health outcomes. More importantly, very few of the studies included other potentially confounding factors such as the timing and effectiveness of public health interventions and other behaviors linked to increased or decreased risk of exposure to the virus. Although more challenging to capture and measure, these public health and behavioral factors play a substantial role in the transmission of the virus (Jüni et al., 2020). Failure to account for public health interventions and behavioral patterns in a statistical analysis was a common limitation across these early studies, and excluding these confounders may contribute to a conclusion that meteorological and air quality factors have a stronger influence on COVID‐19 than is actually the case at this stage of the pandemic. Even fewer studies combined more rigorous statistical analyses and potential confounding factors, which would improve the accuracy of study results.

This review has a number of limitations. It has examined a limited number of studies from a limited phase of the pandemic. Varied data sources were used, and in many cases, the exact sources of data could not be ascertained from the methods as written. While conducting the descriptive analysis, the authors faced challenges in categorizing the methodologies used in the studies. This was often a result of the studies' poor explanation of methods and analyses in addition to a lack of definition of terms or standardized terminology, as discussed previously. Finally, this descriptive analysis was completed by three different reviewers (three authors of this study), which could have been a source of information bias.

## 5 Conclusion

In summary, this descriptive analysis provides an update on previous literature reviews examining the influence of meteorological and air quality factors on COVID‐19 transmission. Similar to previous reviews, this review identifies temperature and humidity as the most common meteorological variables associated with an increase in COVID‐19 transmission. However, heterogeneity and frequent failure to employ robust epidemiologic approaches limit the ability to draw conclusions from the current body of published literature. It would be of value to the research community to use consistent terminology and definitions when studying health outcomes. The authors' intent in conducting and publishing this analysis has been to identify methods and research approaches that are more likely to produce valid results. Although this descriptive review does not follow the strictest standards of a systematic review, the overall recommendations to the research community merit sharing. Despite the challenges of collecting the requisite data, it is important that future studies make stronger efforts to address the issues related to the use of proxy health variables and potentially confounding variables, and to address more explicitly the limitations and uncertainties in the results of these ecological observational studies.

## Conflict of Interest

The authors declare no conflict of interest relevant to this study.

## Supporting information

Data Set S1

## Data Availability

Data are available in the Supporting Information and in a SRDR + repository https://srdrplus.ahrq.gov/public_data?id=977&type=project.
